# Improving detection of tuberculosis among household contacts of index tuberculosis patients by an integrated approach in Myanmar: a cross-sectional study

**DOI:** 10.1186/s12879-018-3586-7

**Published:** 2018-12-14

**Authors:** Kyaw Ko Ko Htet, Tippawan Liabsuetrakul, Saw Thein, Edward B. McNeil, Virasakdi Chongsuvivatwong

**Affiliations:** 1 Department of Medical Research (Pyin Oo Lwin Branch), Pyin Oo Lwin, 100201 Myanmar; 2 Epidemiology Unit, Faculty of Medicine, Prince of Songkla University, Hat Yai, Songkhla, 90110 Thailand; 3 Ministry of Health and Sports, National Tuberculosis Programme, Mandalay, 100107 Myanmar

**Keywords:** Tuberculosis, Screening, Case detection, Determinants, Contact tracing

## Abstract

**Background:**

Contact tracing for tuberculosis (TB) is a recommended measure to improve the case detection rate; however, actual implementation in Myanmar is limited and low detection rates have been reported. Household contacts of a known index TB case are at high risk of infection, thus a more strategic action for contact tracing is required to achieve the goal of the World Health Organization End TB Strategy. This study aimed to assess TB case detection rates among household contacts by an integrated approach and identify risk factors for TB.

**Methods:**

A cross-sectional study was conducted in Mandalay City, Myanmar. Household contacts of index TB cases who had been receiving treatment for at least 3 months were prospectively investigated by an integrated approach which included modification of screening methods and active facilitation of screening investigations as follows. Initial chest x-ray (CXR) was performed for all contacts at the responsible facilities followed by sputum specimen collection for those aged ≥15 years and gene Xpert MTB/RIF examination. Transportation of all household contacts to health facilities and transportation of sputum samples for smear and gene Xpert MTB/RIF examination at centers were arranged by the research team to ensure that all household contacts received all investigations. Risk factors for TB among household contacts were identified by multiple logistic regression models.

**Results:**

Of 174 household contacts, 115 were ≥ 15 years and 59 were < 15 years. The percentage of TB cases detected among the household contacts was 13.8%. There were 14 (12.2%) positive TB cases among the 115 contacts aged ≥15 years while 10 (16.9%) of those aged < 15 years had clinical signs and symptoms of TB with an abnormal CXR. Risk factors among household contacts for TB were being a caretaker of an index case, active and passive smoking, and drinking alcohol.

**Conclusions:**

The integrated approach of TB contact tracing by special arrangement for CXR, sputum and gene Xpert MTB/RIF examination yielded a high TB detection rate in a high TB prevalence area. Logistic and financial administration is needed to strengthen contact tracing. Further research on high-risk household contacts should be considered for increasing TB detection rates.

## Background

Tuberculosis (TB) remains one of the top common infectious diseases, which is a global concern. The World Health Organization (WHO) estimated that two-fifths of new TB cases are undiagnosed and more country-specific actions are needed to identify these missing cases to achieve the global goal of ending the TB epidemic by 2035 [1]. Contact tracing among household family members is one of the active case-finding strategies that has been proposed to increase the case detection rate [2]. In 95 studies from low- and middle-income settings included in a systematic review, the prevalence of TB from household contact tracing varied from 2.1 to 10.1% [3, 4]. Efforts for early diagnosis of TB through effective case finding programs are needed to reduce the rate of TB transmission and to reduce the case detection gap.

Due to the limitations of current passive case-finding strategies and the global urgency to improve TB case-detection rates, WHO has called for more evidence on innovative ways of TB screening, especially from low-income countries. An integrated approach among individuals who are presumed to have TB but do not present to a health service (as commonly seen in the passive case finding approach), has gained interest to enhance the detection rate for early diagnosis and treatment of TB in high prevalence countries [5].

Myanmar is one of the 30 high TB burden countries with an estimated incidence of 365 TB cases per 100,000 population and a high death rate of 49 per 100,000 population in 2015 [1]. For contact investigations, WHO recommends the use of signs and symptoms consistent with TB followed by either acid-fast bacilli (AFB) smear or Chest X Ray (CXR) for contacts aged 10 years or above and less than 10 years, respectively [6]. However, the national guideline of the National Tuberculosis Programme (NTP), Myanmar has applied a diagnosis of TB for those aged < 15 years with signs and symptoms of TB based on CXR due to difficulty in performing the gastric lavage for sputum specimens. In addition, the community-based active case finding through community mobilization, symptom screening and referral, and contact tracing is recommended and the Gene Xpert Mycobacterium tuberculosis/Rifampicin (gene Xpert MTB/RIF examination) is performed in the contacts of multi-drug resistance TB, TB/HIV co-infected patients, TB defaulters and smear positive TB cases [7].

Although the policy of contact tracing has been adopted in Myanmar, there are still constraints and barriers to actually implement and improve TB case detection among household contacts. Therefore, this study aimed to assess TB case detection rates from an integrated approach using a modification of screening methods and active facilitation of screening investigations in Myanmar. Risk factors for TB among household contacts were also identified.

## Methods

### Study design and setting

A cross-sectional study was conducted in Mandalay City, Myanmar from September 2015 to February 2016. Mandalay City is a densely populated area located in the central region of Myanmar containing seven township health departments (THD). TB prevention and control is mainly based on a passive, not active, case finding strategy including screening of TB signs and symptoms followed by CXR among contacts aged < 15 years and sputum examination with CXR among household contacts aged ≥15 years. The community health personnel at each health facility of THD is responsible for TB screening and collection of sputum specimens. The heads of each household were informed about the importance of transporting the sputum samples to a health facility and ensuring that their household members have a CXR performed at a responsible health facility. CXR facilities are available at Mandalay General Hospital and THD in Amarapura Township, Mandalay City.

### Study participants and methods

Index pulmonary TB patients who had been receiving treatment for at least 3 months under the NTP were identified. Temporary residents and those who lived alone or in a temple were excluded due to difficulty in identifying household contacts [8]. Those whose household contacts were not available for interview were also excluded. Sample size for household contacts was calculated based on the one-group precision formula. According to the previous prevalence of TB among household contacts of index TB cases estimated to be 0.13 [8], a type I error of 5% and a precision of 5%, at least 174 household contacts were required. With this sample size, we estimated that 60 index TB cases would be sufficient.

The integrated approach included active facilitation of screening investigations as follows. Initial CXR was performed for all contacts regardless of having signs and symptoms at the responsible facilities followed by sputum specimen collection and gene Xpert MTB/RIF examination for those aged ≥15 years as per the national guideline. For active facilitation of investigations in the initial research protocol, the contacts were encouraged to perform CXR at nearby health facilities and the sputum was collected and sent for further examination by the research team rather than the responsible THD personnel.

In the preparatory phase of the study, we observed that no contacts went to have a CXR by their own arrangement. Therefore, transportation to CXR health facilities for household contacts was arranged by the research team as follows. First, the research team drew a road map for transportation arrangement. Second, appointments were made at times that were convenient to the household contacts. Third, a passenger vehicle with a maximum capacity of 25 persons was organized to transport all contacts whose homes were on or near the same route to the nearby health facilities. Fourth, CXRs for all contacts was facilitated at health facilities and afterwards the contacts were taken back home. Facilitation of transportation for the household contacts to radiography facilities and the sputum specimens to examination facilities were considered as the key enablers of improving case detection rates of TB among household contacts. The assumption of this integrated approach is to enhance the feasibility of complete specimen capture and transport to diagnostic facilities.

### Study sampling

The list of index TB cases receiving treatment for at least 3 months was obtained from the township TB register. From this list, the number of index TB cases was estimated and randomly selected using simple random sampling in order to obtain a sufficient number of household contacts. The research team then contacted the health staff to facilitate home visits of the sampled index TB cases. During these home visits, the inclusion and exclusion criteria of the index TB cases were considered. A second round of sampling of index TB cases was performed in order to achieve the required sample size.

### Data collection

The index TB cases and their household contacts were then informed about the study including all investigations required and those who were willing to participate in the study were asked to sign a consent form independently and separately. The index TB cases and their respective household contacts were then interviewed for background and health-related characteristics by face-to-face interviews using a structured questionnaire.

A Garmin global positioning system (GPS) Colorado 300 device was used to record the distance of their location to health facilities [9]. The research team drew a road map and determined the closest responsible CXR facility based on the GPS and then arranged group transportation for the household contacts living in the same areas to the closest health facility. After the community health personnel in each assigned area collected the sputum specimen from household contacts, the research team transported the sputum specimens to the health facility for AFB or gene Xpert MTB/RIF examination. All of the above steps above were carried out prospectively. The results of all screening tests were interpreted by the responsible providers independently and blindingly.

### Variables

The outcome variable was operationally defined as the positive TB detection of household contacts which was classified as one of the following conditions: (1) abnormal CXR suggestive of TB and the presence of any signs and symptoms of suspected TB among the household contacts aged < 15 years or along with positive sputum AFB or gene Xpert MTB/RIF assay among the household contacts aged ≥15 years, or (2) positive sputum with the presence of at least one AFB in at least one sputum sample among the household contacts aged ≥15 years. Contacts aged < 15 years without abnormal CXR finding were treated with antibiotics and observed for clinical response for at least two weeks according to the national guideline if they had one or more of the following symptoms: cough lasting 2 weeks or more, blood in sputum, fever lasting more than 2 weeks, weight loss and chest pain. The same treatment was given to the contacts aged ≥15 years who had abnormal CXR without confirmation with sputum AFB or gene Xpert MTB/RIF examinations.

Independent variables included background characteristics such as gender, age, ethnicity, education level, occupation, marital status, daily income, living alone in a separate room, number of persons living together, sharing a room with an index TB case and caretaker to index TB case, and health related risky characteristics such as active smoking status (never, former or current), smoking duration and intensity, passive smoking status, drinking status (never, former or current) and presence of any signs and symptoms of TB. Point coordinate data for location was collected as longitude and latitude to measure distance between patient’s residences to the nearest CXR center.

### Statistical analysis

All data were entered into EpiData and analyzed using R (2013 The R Foundation for Statistical Computing). The characteristics and information of index TB cases and their household contacts were presented descriptively using frequencies and percentages. The determinants of positive TB among household contacts were initially explored using univariate analysis and the variables having a *p*-value less than 0.2 were included in the initial multivariate logistic regression model. Variables having a *p-*value of < 0.05 were kept in the final model and considered as statistically significant.

## Results

**Fig. 1:**
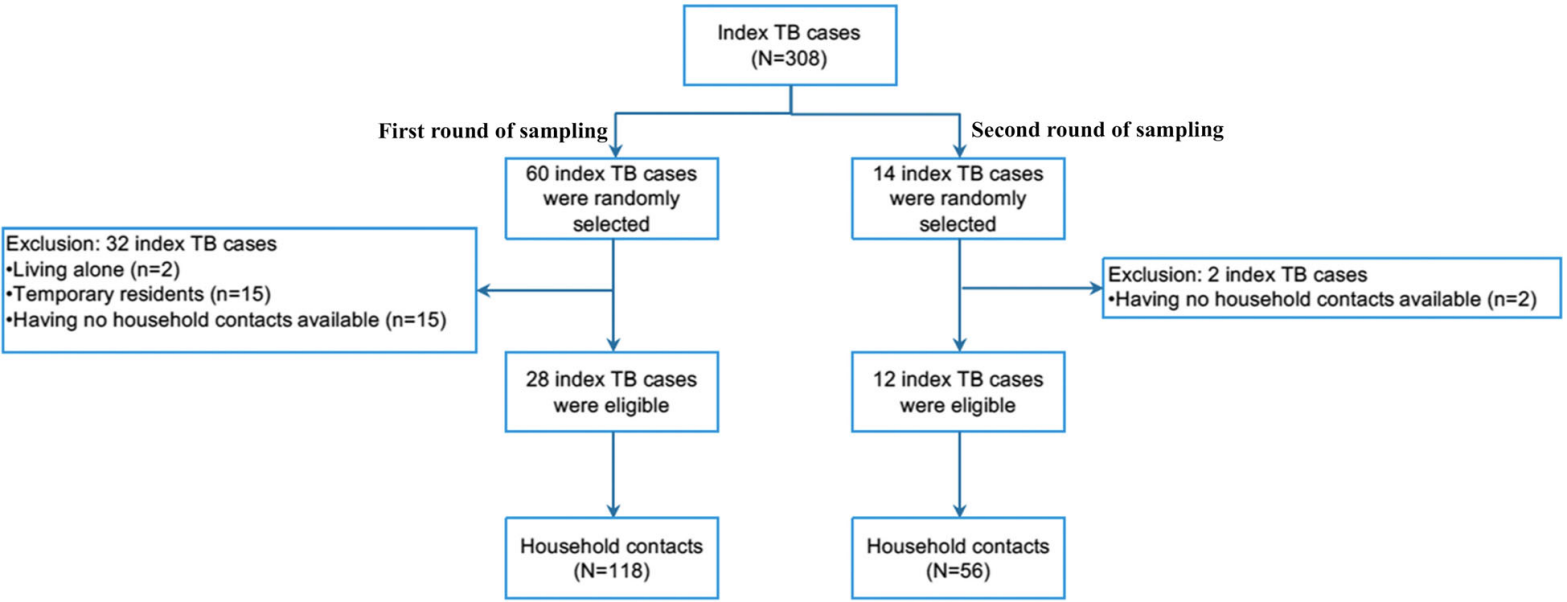
Diagram flow of study sampling for index TB cases

Of 308 index TB cases identified, 40 were included in the study together with 174 household contacts from two rounds of sampling. The diagram flow of study sampling is shown in Fig. 1. There were 34 index TB cases excluded due to living alone (*n* = 2), temporary resident (*n* = 15), and having no household contacts available (*n* = 17). The median duration of TB treatment was 4.1 months (Interquartile range - IQR: 3.2–4.8).

Tables 1 and 2 present the background and health-related risk characteristics of the study index TB cases and household contacts, respectively. Males accounted for > 70% of index TB cases and 40% of household contacts. The mean ± standard deviation (SD) age was 34.6 ± 17.9 years for index TB cases and 28.9 ± 19 years for household contacts. Of the 174 contacts, 115 were aged ≥15 years (median age = 9, IQR 6–10) and 59 were aged < 15 years (median age = 38, IQR 29–50). Contacts aged under five years accounted for 4.6%. The average number of household contacts in a family was 4.0 (SD = 1.9, range 2–10). Among TB index cases, 30% were former or current smokers while the rate among household contacts was 18.4%. The prevalence of former or current drinking was 15% for both groups. Approximately 44% of contacts had signs and symptoms of TB.

**Table 1:** Background characteristics of index TB cases and household contacts

Variable	Index TB cases		Household contacts	
	(*n* = 40)		(*n* = 174)	
	n	(%)	n	(%)
Gender				
Male	29	(72.5)	71	(40.8)
Female	11	(27.5)	103	(59.2)
Age (years)				
< 15	7	(17.5)	59	(33.9)
≥15	33	(82.5)	115	(66.1)
Education				
None	15	(37.5)	57	(32.8)
Primary School	16	(40.0)	62	(35.6)
Middle School	3	(7.5)	38	(21.8)
High/Graduate School	6	(15.0)	17	(9.8)
Occupation				
Factory worker	12	(30.0)	47	(27.0)
Casual employee	12	(30.0)	37	(21.3)
Dependent/student	16	(40.0)	90	(51.7)
Marital Status				
Single	12	(30.0)	89	(51.1)
Married	28	(70.0)	85	(48.9)

*TB* tuberculosis

**Table 2:** Health related risky characteristics of index TB cases and household contacts

Variable	Index TB cases		Household contacts	
	(*n* = 40)		(*n* = 174)	
	n	(%)	n	(%)
Smoking status				
Never smoker	28	(70.0)	142	(81.6)
Former or current smoker	12	(30.0)	32	(18.4)
Number of cigarettes smoked per day among former or current smokers				
Mean (SD)	3.8	(1.0)	2.9	(1.9)
Duration of smoking (years) among former or current smokers				
< 20	6	(50.0)	20	(62.5)
≥ 20	6	(50.0)	12	(37.5)
Passive smoking status				
No	35	(87.5)	115	(66.1)
Yes	5	(12.5)	59	(33.9)
Drinking status				
Never drinker	34	(85.0)	149	(85.6)
Former or current drinker	6	(15.0)	25	(14.4)
Duration of drinking (years) among former or current drinkers				
Mean (SD)	9.6	(3.4)	6.3	(6.1)

*TB* tuberculosis, *SD* standard deviation

Of the 174 household contacts, abnormal CXR suggestive of TB was found in 27 contacts, of which 17 and 10 were aged ≥15 years and < 15 years, respectively. Among the 17 contacts aged ≥15 years with abnormal CXR, 9 were confirmed positive for TB by AFB, 5 by gene Xpert MTB/RIF examination, and 3 were negative, all of which were followed up by the responsible THD. The overall rate of positive TB detection among household contacts was 13.8%, of which 12.2% and 16.9% were detected among contacts aged ≥15 years (*n* = 14/115) and those aged < 15 years (*n* = 10/59), respectively. The distance from contacts’ home to the responsible CXR facility at Amarapura township ranged from 1.1 to 9.7 km and at Mandalay General Hospital ranged from 2.9 to 17.5 km.

Table 3 shows results of the univariate analysis for identifying determinants of positive TB among household contacts. Significant factors were male gender, sharing a room with an index TB case, being a caretaker to an index TB case, current or passive smoker, and current or former drinker. Table 4 shows the results of the final multiple logistic regression model for predicting TB among household contacts. Significant risk factors were being caretaker of an index TB case, active smoker, passive smoker, and former or current drinker (Table 4).

**Table 3:** Comparison of background and health related characteristics among household contacts by TB status

	TB status among household contacts				
Variable	Negative		Positive		*P* value
	(*n* = 150)		(*n* = 24)		
	n	(%)	n	(%)	
Gender					0.003
Male	54	(36.0)	17	(66.7)	
Female	96	(64.0)	7	(33.3)	
Age (years)					0.53
< 15	49	(32.7)	10	(41.7)	
≥15	101	(67.3)	14	(58.3)	
Ethnicity					0.72
Burmese	135	(90.0)	21	(87.5)	
Others	15	(10.0)	3	(12.5)	
Education					0.24
≤ Primary	100	(66.7)	19	(79.2)	
> Primary	50	(33.3)	5	(20.8)	
Occupation					0.96
Factory worker	41	(27.3)	6	(25.0)	
Casual employee	32	(21.3)	5	(20.8)	
Dependent/student	77	(51.4)	13	(54.2)	
Marital status					0.73
Single	78	(52.0)	11	(45.8)	
Married	72	(48.0)	13	(54.2)	
Daily income (USD)					0.32
< 3	132	(88.0)	19	(79.2)	
≥ 3	18	(12.0)	5	(20.8)	
Living alone in separate room					0.76
No	128	(85.3)	20	(83.3)	
Yes	22	(14.7)	4	(16.7)	
Number of persons living together					0.601
≤ 3	87	(58.0)	12	(50.0)	
> 3	63	(42.0)	12	(50.0)	
Sharing room with a TB patient					0.002
No	87	(58.0)	5	(20.8)	
Yes	63	(42.0)	19	(79.2)	
Caretaker to index TB case					< 0.001
No	116	(77.3)	7	(29.2)	
Yes	34	(22.7)	17	(70.8)	
Smoking status					< 0.001
Never smoker	132	(88.0)	10	(41.7)	
Former or current smoker	18	(12.0)	14	(58.3)	
Number of cigarettes smoked per day among smokers					0.71
< 4	13	(72.2)	9	(64.3)	
≥ 4	5	(27.8)	5	(35.7)	
Duration of smoking (years) among smokers					1
< 20	11	(61.1)	9	(64.3)	
≥ 20	7	(38.9)	5	(35.7)	
Passive smoking status					0.003
No	106	(70.7)	9	(37.5)	
Yes	44	(29.3)	15	(62.5)	
Drinking status					< 0.001
Never drinker	138	(92.0)	11	(45.8)	
Former or current drinker	12	(8.0)	13	(54.2)	
Duration of drinking (years) (*n* = 25)					0.67
< 7	9	(76.9)	8	(58.3)	
≥ 7	3	(23.1)	5	(41.7)	
Signs and symptoms of TB					0.69
No	86	(57.3)	11	(45.8)	
Yes	64	(42.7)	13	(54.2)	

*TB* Tuberculosis, *USD* United States dollars

**Table 4:** Factors associated with positive TB among household contacts

Variable	Crude OR (95% CI)	Adjusted OR (95% CI)	*P* value^†^
Caretaker to index TB case			< 0.001
No	1	1	
Yes	8.3 (3.17–21.6)	10.4 (3.09–35.0)	
Active smoking status			0.044
Never smoker	1	1	
Former or current smoker	10.3 (3.97–26.5)	6.4 (1.42–28.5)	
Passive smoking status			0.017
No	1	1	
Yes	4.0 (1.64–9.9)	4.0 (1.28–12.7)	
Drinking status			0.015
Never drinker	1	1	
Former or current drinker	13.6 (5.02–36.8)	4.6 (1.02–20.8)	

^†^Likelihood ratio test

*OR* odds ratio, *CI* confidence interval, *TB* Tuberculosis

## Discussion

The integrated approach of TB contact tracing by special arrangement for CXR, sputum and Gene Xpert examination yielded a high rate of TB detection among household contacts in a high TB prevalence area. The determinants of positive TB among household contacts were being a caretaker of an index TB case, active and passive smoking, and former or current alcohol drinking. Without facilitation for transportation or a voluntary request that household contacts have a sputum test and CXR, the policy of household contact tracing is not feasible in Mandalay City.

The percentage of positive TB cases among household contacts in our study was higher than studies from China (3.8%) and Nepal (1.6%) [10, 11] and similar to studies from South Africa (13.8%) and India (10.1%) [3, 8]. The differences of case detection among household contacts can be explained by the use of comprehensive strategies to obtain the contacts, different measurement tools, or the nature of living styles in each country [12]. Apart from the National Tuberculosis Programme, Myanmar, international non-governmental organizations have also contributed to the activities of active case finding ranging from 15 to 52%, while the local non-governmental organizations contributed around 4 to 6% [13, 14]. Moreover, the integrated approach, which included three screening methods of diagnosis, namely CXR, sputum smear, and Xpert MTB/RIF, could have increased the rate of high TB case detection.

From previous studies, a low detection rate of 0.2 to 6.2% was found when TB signs and symptoms was used for initial screening [15], while high sensitivity of CXR to detect pulmonary TB was reported in both adults and children [16]. For the challenge of completing the investigations, active facilitation of contacts’ transportation for CXR investigation and sputum examination introduced in our study could be helpful to overcome the financial hardship, especially on the indirect costs which was a common barrier for contact investigations [17]. One study in a community-based active TB care facility in Myanmar also revealed that transportation fees for sputum samples are inadequately covered by the NTP and are a probable barrier for improving TB case contribution [14]. This is consistent with the consideration of economic barriers and reducing the out-of-pocket expenses of TB patients and their contacts in the Global Fund to Fight AIDS, Tuberculosis and Malaria [18].

Being a caretaker of an index TB case has been shown to be a strong determinant of positive TB among contacts [19]. This may be explained by close exposure of infectious droplets from index cases without having proper protection [20]. The intensive education and practices on source control techniques, including cough etiquette and respiratory hygiene, spending time outside with good ventilation, and sleeping in separate rooms, have been shown to be important for preventing the spread of TB to household contacts [21]. Smoking and passive smoking can cause pathological changes in lung function and drinking alcohol can decrease the immune system’s ability to fight infection [22]. Gender was found to be associated with positive TB [23]. Males are more likely to access a health center for confirming TB diagnosis where a passive case finding is used. In our study, we conducted active case finding and the gender ratio was balanced. Our results showed that gender was not a significant determinant for positive TB among contacts.

WHO has recently released chest radiography guidelines that recommend it as the preferred TB screening tool from the viewpoint of test accuracy, noting that it can be expensive and logistically challenging to use [24]. The findings of our integrated approach, which applied initial CXR with active facilitation of transport for contact investigations in both CXR and sputum examinations, will be useful to other similar resource-limited settings where TB is endemic. With this approach, a high TB detection rate among household contacts was assured as we conducted a prospectively active contact tracing study, thus reducing under-estimation of TB infection comparing to routine or passive case findings.

There were some limitations in this study. First, the exclusion of index TB cases if all of their household contacts were not available may have led to selection bias of the households with healthy contacts working out of the hometown. However, this was unavoidable and may not be relevant to the definition of household contacts. Second, both CXR and sputum testing could be collected only in contacts aged 15 years or more. Third, extra pulmonary TB and latent TB infections among contacts were not detected. Fourth, genotype matching was not performed, thus we could not ascertain whether or not the contacts were infected by the index TB cases living in the same household. Finally, active facilitation of transportation of household contacts for CXR and sputum investigation used in our study may not be generalized in real practice settings. However, this active facilitation of transportation can be considered in the strategic planning for other low-income countries where TB populations face financial difficulty in access to TB investigations and out-of-pocket transportation costs.

## Conclusions

This study highlights a high rate of TB detection by an integrated approach. Household contacts who were caretakers of a TB patient, active and passive smokers, and former or current drinkers were high-risk groups. Transportation and financial relief should be considered and prioritized to strengthen the contact tracing strategy of the country. The appropriate amount of funding for TB prevention and control among household contacts needs to be determined.

## Abbreviations

AFB: Acid-fast bacilli
CXR: Chest x-ray
Gene Xpert MTB/RIF: Gene Xpert Mycobacterium tuberculosis/Rifampicin
GPS: Global positioning system
IQR: Interquartile range
NTP: National Tuberculosis Programme
SD: Standard deviation
TB: Tuberculosis
THD: Township health departments
WHO: World Health Organization
